# Unveiling the Different Chemical Reactivity of Diphenyl Nitrilimine and Phenyl Nitrile Oxide in [3+2] Cycloaddition Reactions with (R)-Carvone through the Molecular Electron Density Theory

**DOI:** 10.3390/molecules25051085

**Published:** 2020-02-28

**Authors:** Mar Ríos-Gutiérrez, Luis R. Domingo, M’hamed Esseffar, Ali Oubella, My Youssef Ait Itto

**Affiliations:** 1 Department of Organic Chemistry, University of Valencia, Dr. Moliner 50, Burjassot, E-46100 Valencia, Spain; domingo@utopia.uv.es; 2 Department of Chemistry and Chemical Biology, McMaster University, 1280 Main Street West, Hamilton, Ontario L8S 4L8, Canada; 3 Laboratoire de Synthèse Organique et Physico-Chimie Moléculaire, Département de Chimie, Faculté des Sciences, Semlalia, B.P 2390, Marrakech 40001, Morocco; esseffar@uca.ac.ma (M.E.); oubellaali1@gmail.com (A.O.); aititto@uca.ac.ma (M.Y.A.I.)

**Keywords:** [3+2] cycloadditions, nitrilimines, nitrile oxides, chemoselectivity, molecular electron density theory, molecular mechanisms

## Abstract

The [3+2] cycloaddition (32CA) reactions of diphenyl nitrilimine and phenyl nitrile oxide with (R)-carvone have been studied within the Molecular Electron Density Theory (MEDT). Electron localisation function (ELF) analysis of these three-atom-components (TACs) permits its characterisation as carbenoid and zwitterionic TACs, thus having a different reactivity. The analysis of the conceptual Density Functional Theory (DFT) indices accounts for the very low polar character of these 32CA reactions, while analysis of the DFT energies accounts for the opposite chemoselectivity experimentally observed. Topological analysis of the ELF along the single bond formation makes it possible to characterise the mechanisms of these 32CA reactions as *cb-* and *zw-type.* The present MEDT study supports the proposed classification of 32CA reactions into *pdr-*, *pmr-*, *cb-* and *zw-type*, thus asserting MEDT as the theory able to explain chemical reactivity in Organic Chemistry.

## 1. Introduction

Five membered heterocyclic compounds, particularly those having the pyrazole and isoxazole frameworks, are components of a diverse array of compounds with a broad spectrum of bioactivities such as analgesic, [1,2,3] anti-inflammatory [3,4,5], antibacterial [3,6,7], antifungal [3,8,9], antitumoral [3,10,11] and antiviral [3,12,13] activities.

Among the synthetic routes most often employed, the [3+2] cycloaddition (32CA) reaction of the corresponding three-atom-component (TAC) with an appropriate ethylene derivative is the main approach to access these valuable skeletons, because of its simplicity, efficiency and high selectivity [10,14,15].

**Scheme 1:**
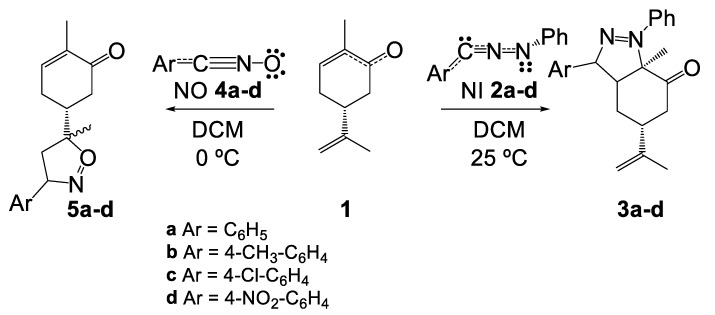
[3+2] cycloaddition (32CA) reactions of (R)-carvone **1** with diaryl NIs **2a–d** and aryl-NOs **4a–d**.

Very recently, Ait Itto et al. reported the 32CA reactions of (R)-carvone **1** with diaryl nitrilimines (NIs) **2a–d** and arylnitrile oxides (NOs) **4a–d** in dichloromethane (DCM), yielding pyrazoles **3a–d** and isoxazoles **5a–r**, respectively (see Scheme 1) [16]. These 32CA reactions presented total regio- and chemoselectivity, resulting in a mixture of a pair of diastereoisomers resulting from the attack of these TACs by the two faces of the two C−C double bonds of (R)-carvone **1**. Interestingly, these 32CA reactions showed contrary chemoselectivity depending on the nature of the TAC; thus, diaryl NIs **2a–d** preferred the attack on the *α*,*β*-conjugated C−C double bond of (R)-carvone **1**, while aryl-NOs **4a–d** preferred the attack on the isopropenyl C−C double bond (see Scheme 1) [16].

The study of chemical reactivity is incomplete without the blending of experimental results and theoretical rationalisation. 32CA reactions have been widely studied theoretically. In 2007, Houk proposed the Distortion/Interaction Model (DIM) [17,18], which is analogous to the Activation Strain Model (ASM) proposed in 1999 by Bickelhaupt [19], to explain the reactivity of the twelve simplest TACs in 32CA reactions towards ethylene **6** and acetylene **7**. Houk found that the computed B3LYP/6-31G(d) activation enthalpies correlated very nicely with the distortion energies ΔE_d_^≠^. However, in spite of this good correlation between the activation enthalpies and the distortion energies ΔE_d_^≠^, that energy decomposition analysis was not able to explain the different experimentally observed chemical reactivity of the studied TACs.

Recent advances made in the theoretical understanding of 32CA reactions based on the Molecular Electron Density Theory [20] (MEDT) have allowed establishing a very good correlation between the electronic structure of the simplest TACs and their reactivity towards ethylene **6 [21]**. Accordingly, depending on the electronic structure of the simplest TACs, *pseudodiradical*, *pseudoradical*, carbenoid and zwitterionic, 32CA reactions have been classified into *pdr-type*, *pmr-type*, *cb-type* and *zw-type* reactions [21], respectively, in such a manner that while *pdr-type* 32CA reactions can be carried out very easily, *zw-type* 32CA reactions demand adequate nucleophilic/electrophilic activations to take place [22].

**Chart 1:**
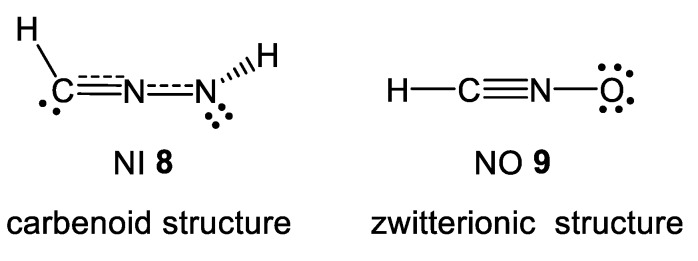
Electron Localisation Function (ELF)-based carbenoid and zwitterionic Lewis-like structures of the simplest NI **8** and NO **9** Three-Atom-Components (TACs).

These MEDT studies permitted the classification of the simplest NI **8** as a carbenoid TAC [23] and the simplest NO **9** as a zwitterionic TAC [24] (see Chart 1), also characterising a different reactivity for both of them. Thus, while carbenoid NI **8** reacts through the nucleophilic attack on the *β*-conjugated position of electrophilic ethylene in a *cb-type* 32CA reaction [23], the low electrophilic and nucleophilic character of zwitterionic NO **9** makes it participate in *zw-type* 32CA reactions with low polar character and high activation energies [24]. Consequently, it is expected that the aryl-substituted NIs **2a–d** and NOs **4a–d** given in Scheme 1 would experience a different reactivity towards the *α*,*β*-conjugated and the isopropenyl C−C double bonds present in (R)-carvone **1**.

Thus, in view of the different chemical reactivity experienced by diaryl-NIs **2a–d** and aryl-NOs **4a–d** towards the two different C−C double bonds of (R)-carvone **1**, experimentally studied by Ait Itto et al. [16], an MEDT study of the 32CA reactions of diphenyl NI **2a** and with phenyl-NO **4a** towards (R)-carvone **1** is herein carried out in order to shed light on the experimental outcomes, i.e., the opposite chemoselectivity of the 32CA reactions of these TACs with (R)-carvone **1**.

## 2. Results and Discussion

### 2.1. Topological Analysis of the Electron Localisation Function (ELF) and Natural Population Analysis (NPA) of Diphenyl-NI ***2a***, Phenyl-NO ***4a*** and (R)-Carvone ***1***

**Figure 1:**
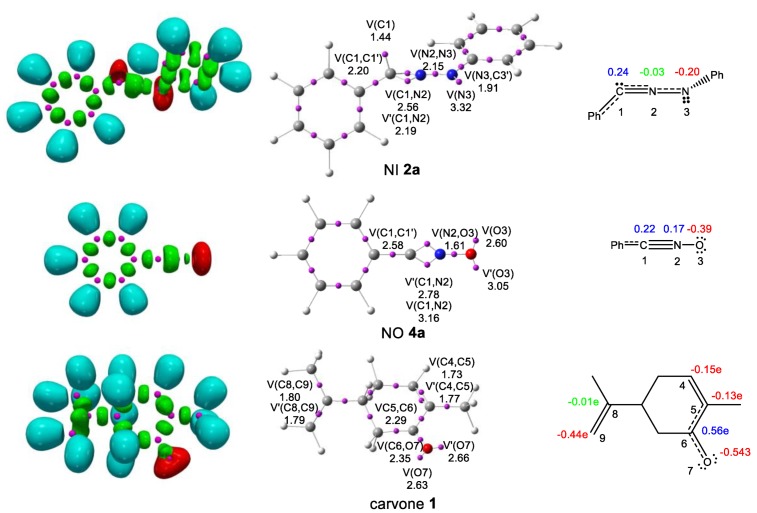
B3LYP/6-31G(d) ELF localisation domains, represented at an isosurface value of ELF = 0.75, ELF basin attractor positions, together with the valence basin populations, and the proposed ELF-based Lewis-like structures, together with the natural atomic charges, of diphenyl-NI **2a**, phenyl-NO **4a** and (R)-carvone **1**. For ELF localisation domains, protonated domains are coloured in turquoise, disynaptic domains in green, monosynaptic domains in red, and core domains in purple. For the geometries, the standard colour code for chemical elements is used, while the attractors of ELF basins are coloured in purple. For charges, negative charges are coloured in red, positive charges in blue and negligible charges in green. ELF valence basin populations and natural atomic charges are given in average number of electrons, e.

In order to characterise the electronic structure of diphenyl-NI **2a** and phenyl NO **4a** and, thus, to establish their reactivity in 32CA reactions [21], a topological analysis of the ELF [25] of these TACs was first performed; the electronic structure of (R)-carvone **1** is also analysed. ELF localisation domains, ELF basin attractor positions together with the valence basin populations, as well as the proposed ELF-based Lewis-like structures together with the natural atomic charges, are shown in Figure 1. Lewis-like structures come from the adaption of ELF basin populations to fit the Lewis bonding model. Thus, populations below 1e have been associated with radical or *pseudoradical* centres, populations between 1e and 2e with a single bond or a lone pair, between 3e and 4e with double bonds or two lone pairs, while more than 5e are associated with triple bonds or three lone pairs. 

Topological analysis of the ELF of diphenyl NI **2a** shows the presence of one monosynaptic basin V(C1) integrating 1.44 e, two disynaptic basins, V(C1,N2) and V’(C1,N2), integrating 2.56 e and 2.19 e, one V(N2,N3) disynaptic basin integrating 2.15 e, and one V(N3) monosynaptic basin integrating 3.32e. Within the context of ELF, monosynaptic basins, labelled V(A), are associated with non-bonding electron density regions, while disynaptic basins, labelled V(A,B), connect the core of two nuclei A and B and, thus, correspond to a bonding region between nuclei A and B [26]. This description, together with the ELF valence basin populations, allows relating the monosynaptic basin V(C1) with a depopulated C1 carbenoid centre, the two disynaptic basins, V(C1,N2) and V’(C1,N2), with a populated C1−N2 double bond, integrating a total of 4.75 e, the V(N2,N3) disynaptic basin with a slightly overpopulated N2−N3 single bond, and the V(N3) monosynaptic basin with an overpopulated N3 lone pair. The non-planar geometry of diphenyl NI **2a**, together with the presence of the carbenoid centre, suggest that this linear TAC has an allenic structure [23]. Consequently, the topological analysis of the ELF of diphenyl NI **2a** indicated that, just as the simplest NI **8 [23]**, this TAC has a carbenoid structure participating in *cb-type* 32CA reactions.

Topological analysis of the ELF of phenyl NO **4a** shows the presence of two disynaptic basins, V(C1,N2) and V’(C1,N2), integrating 2.78 e and 3.16 e, one V(N2,O3) disynaptic basin integrating 1.61 e, and two monosynaptic basins, V(O3) and V’(O3) integrating 2.60 e and 3.05 e. The sinapticity of the valence basins, together with the ELF valence basin populations, allows relating the V(C1,N2) and V’(C1,N2) disynaptic basins, which integrate a total of 5.94 e, with a C−N triple bond, the V(N2,O3) disynaptic basin with a depopulated N2−O3 single bond, and the two V(O3) and V’(O3) monosynaptic basins with two overpopulated O3 lone pairs. The linear geometry of phenyl NO **9,** together with this ELF analysis suggest that this TAC has a propargylic structure. Consequently, the topological analysis of the ELF of phenyl NO **4a** indicates that this TAC has a zwitterionic structure [24] participating in *zw-type* 32CA reactions, just as the simplest NO **9**, thus presenting a different reactivity to that of carbenoid diphenyl NI **2a**.

Finally, topological analysis of the ELF of (R)-carvone **1** shows the presence of two disynaptic basins, V(C4,C5) and V’(C4,C5), integrating 1.77 e and 1.76 e, one V(C5,C6) disynaptic basin integrating 2.29 e, one V(C6,O7) disynaptic basin integrating 2.35 e, and two monosynaptic basins, V(O7) and V’(O7), integrating 2.66 e and 2.63 e. On the other hand, (R)-carvone **1** also shows the presence of two disynaptic basins, V(C8,C9) and V’(C8,C9), integrating 1.80 e and 1.79 e. The sinapticity of the valence basins, together with the ELF valence basin populations, allows relating the V(C4,C5) and V’(C4,C5) disynaptic basins with a C4−C5 double bond, integrating a total of 3.53 e, the V(C5,C6) and V(C6,O7) disynaptic basins to a populated C5–C6 and C6-O7 single bonds, and the two V(O7) and V’(O7) monosynaptic basins with two overpopulated O7 lone pairs. Finally, the two V(C8,C9) and V’(C8,C9) disynaptic basins could be related to a C8-C9 double bond.

Once the bonding patterns of the reagents were established, the charge distribution was analysed through natural population analysis (NPA). NPA of diphenyl NI **2a** and phenyl NO **4a** shows polarisation of the electron density of these TACs towards the terminal N3 nitrogen and O3 oxygen, respectively. Note that this polarisation is larger at phenyl NO **4a** as a consequence of the more electronegative character of the oxygen atom that the nitrogen one (see Figure 1). It is interesting to remark that the charge distribution of phenyl NO **4a**, which is classified as a zwitterionic TAC, is not a consequence of the set of resonance Lewis structures, as proposed [27], but of the different nature of the three nuclei of this TAC [21].

### 2.2. Analysis of the Conceptual Density Functional (CDFT) Reactivity Indices of the Reagents

Numerous studies devoted to Diels-Alder and 32CA reactions have shown that the analysis of the reactivity indices defined within CDFT [28,29] is a powerful tool to predict and understand the reactivity in polar and ionic organic reactions. Thus, in order to predict the reactivity of diphenyl NI **2a**, phenyl NO **4a** and (R)-carvone **1** in the corresponding 32CA reactions, the global CDFT reactivity indices, i.e., the electronic chemical potential, μ, the chemical hardness, η, the electrophilicity, ω, and the nucleophilicity, *N*, at the ground state of the reagents, were computed and analysed (see Table 1).

The electronic chemical potential μ [30,31] of diphenyl NI **2a**, μ = −3.36 eV, is only slightly higher than that of (R)-carvone**1**, μ = −3.84 eV; consequently, diphenyl NI **2a** will not be very prone to transfer electron density towards (R)-carvone **1**, and the corresponding 32CA reaction is expected to have a low polar character. On the order hand, the electronic chemical potential μ of the phenyl NO **4a**, μ = −3.83 eV, is almost identical to that of (R)-carvone **1**, indicating that the corresponding 32CA reaction will have a clear non-polar character.

The chemical hardness η indices of the simplest NI **8** and NO **9** are 5.87 and 7.94 eV. The substitution of the hydrogens by phenyl groups decreases by ca. 2 eV the chemical hardness η [30] of both experimental TACs diphenyl NI **2a** and phenyl NO **4a** (see Table 1), indicating that the substitution makes them more prone to the bonding changes, thus decreasing the activation energies. The chemical hardness η of diphenyl NI **2a**, 3.78 eV, is much lower than that of (R)-carvone **1**, 5.23 eV, indicating that along the reaction path, the bonding changes at the NI framework will take place more easily than those at the ethylene one. Conversely, the chemical hardness η of phenyl NO **4a**, 5.02 eV, is similar to that of (R)-carvone **1**, indicating a similar trend for bonding changes in both structures along the reaction path.

The electrophilicity ω index [32] of the simplest NI **8** and the simplest NO **9** is 1.07 and 0.73 eV, respectively, being classified as strong and moderate electrophiles within the electrophilicity scale [29]. On the other hand, the nucleophilicity *N* index [33] of these TACs is 2.64 (**8**) and 1.75 (**9**) eV. Thus, while the simplest NI **8** is classified as a moderate nucleophile, the simplest NO **9** is classified as a marginal nucleophile within the nucleophilicity scale [29]. The low electrophilicity ω and nucleophilicity *N* indices of the simplest NO **9** indicate that the corresponding *zw-type* 32CA reaction will have a non-polar character, in agreement with the analysis of the electronic chemical potentials, and high activation energy [24].

On the other hand, diphenyl NI **2a** and phenyl NO **4a** have similar electrophilicity indices, 1.50 and 1.46 eV, respectively, both being classified as strong electrophiles, while their nucleophilicity *N* indices are 3.87 (**2a**) and 2.78 (**4a**) eV. Thus, while diphenyl NI **2a** is classified as a strong nucleophile, phenyl NO **2a** is classified as a moderate nucleophile. Consequently, the inclusion of the phenyl group in the simplest TACs NI **8** and NO **9** increases their electrophilic and nucleophilic character.

The electrophilicity ω and nucleophilicity *N* indices of (R)-carvone **1** are 1.41 eV and 2.67 eV, respectively, is classified as a moderate electrophile and a moderate nucleophile.

Analysis of the global CDFT indices indicates that although diphenyl NI **2a** is classified as a strong nucleophile, the low electrophilic character of (R)-carvone **1** provides the corresponding 32CA reaction with a very low polar character. On the other hand, the low electrophilic and nucleophilic character of phenyl NO **4a** does not permit its participation in polar processes.

### 2.3. Study of the Reaction Paths Associated with the 32CA Reactions of Diphenyl NI ***2a*** and Phenyl NO ***4a*** with (R)-Carvone ***1***

#### 2.3.1. Study of the 32CA Reactions of Diphenyl NI **2a** with (R)-Carvone **1**

**Scheme 2:**
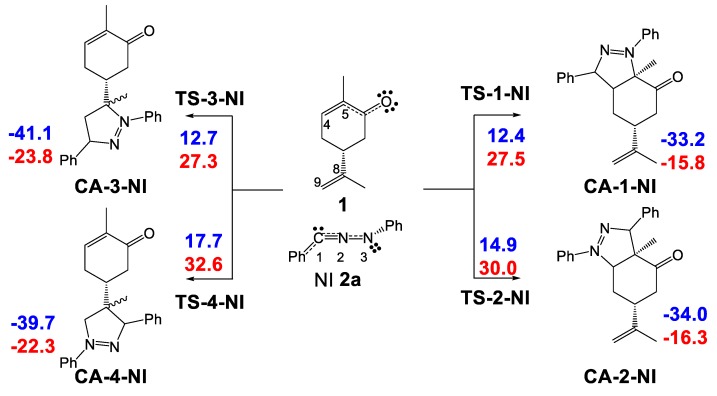
Studied reaction paths associated with the 32CA reaction of diphenyl NI **2a** with (R)-carvone **1**. B3LYP/6-311G(d,p) relative enthalpies, in blue, and relative Gibbs free energies, in red, computed at 25 ˚C in DCM, are given in kcal·moL^−1^. The energy reference is the separated reagents.

First, the 32CA reaction of diphenyl NI **2a** with (R)-carvone **1** was studied. Due to the non-symmetry of the reagents and the presence of two C-C double bonds in (R)-carvone **1**, eight reactive paths are feasible. Only four reaction paths were considered: the attack to the C4−C5 or C8−C9 double bonds of (R)-carvone **1**, and the two regioisomeric approach modes of diphenyl NI **2a** to these C−C double bonds (see Scheme 2). Facial selectivity was not considered [16]. Analysis of the mentioned reaction paths allowed locating and characterising four TSs and the corresponding pyrazoles, indicating that this 32CA reaction takes place through a one-step mechanism (see Scheme 2). B3LYP/6-311G(d,p) relative enthalpies and Gibbs free energies in DCM are given in Scheme 2. The total and relative energies in the gas phase and in DCM, as well as the thermodynamic data, are given in Tables S4 and S7 in the Supplementary Material.

The B3LYP/6-311G(d,p) activation enthalpies associated with the four competitive reaction paths are 12.4 (**TS-1-NI**) 14.9 (**TS-2-NI**), 12.7 (**TS-3-NI**), and 17.7 (**TS4-NI**) kcal·moL^−1^, the formation of the pyrazoles being exothermic by 33–41 kcal·moL^−1^. Some appealing conclusions can be drawn from these energy results: (i) this 32CA reaction presents an activation enthalpy via **TS-1-NI** of 12.4 kcal·moL^−1^. The activation energy associated with the 32CA between the simplest NI **8** and ethylene **6** is 10.5 kcal·moL^−1^ (see Table S8 in Supplementary Material); (ii) this 32CA reaction is highly regioselective, as **TS-2-NI** is 2.5 kcal·moL^−1^ higher in enthalpy than **TS-1-NI**; (iii) this 32CA reaction presents a low chemoselectivity as **TS-3-NI** is only 0.3 kcal·moL^−1^ higher in enthalpy than **TS-1-NI**. Note, however, that at the MPWB1K computational level, **TS-3-NI** is 3.0 kcal·moL^−1^ higher in enthalpy than **TS-1-NI** (see Table S1 in Supplementary Material); (iv) the high exothermic character of the four reaction paths makes them irreversible. Consequently, the formation of the most favourable pyrazole **CA-1-NI** is subject to a kinetic control.

**Figure 2:**
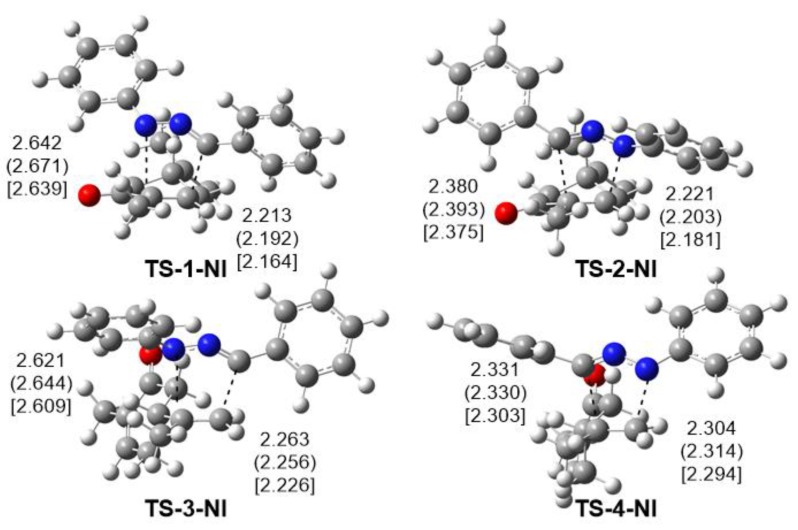
B3LYP/6-31G(d) optimised geometries, in the gas phase and in dichloromethane (DCM), of the transition state structures (TSs) involved in the 32CA reaction of diphenyl NI **2a** with (R)-carvone **1**. Distances are given in angstroms, Å. Values in DCM are given in parentheses, while the B3LYP/6-311G(d,p) values in DCM are given in brackets.

The B3LYP/6-31G(d) gas phase and in DCM geometries of the four TSs are given in Figure 2. The distances between the interacting C1 and Cx and N3 and Cy centres at the gas phase TSs are 2.213 (C1−C4) and 2.642 (N3−C5) Å at **TS-1-NI**, 2.221 (N3−C4) and 2.380 (C1−C5) Å at **TS-2-NI**,2.263 (C1−C8) and 2.621 (N3−C9) Å at **TS-3-NI**, and 2.304 (N3−C8) and 2.331 (C1−C9) Å at **TS-4-NI**. Some appealing conclusions can be drawn from these geometrical parameters: (i) the more favourable regioisomeric TSs,**TS-1-NI** and **TS-3-NI**, are more asynchronous than **TS-2-NI** and **TS-4-NI**; (ii) the most favourable **TS-1-NI** is associated with a two-centre interaction between the most nucleophilic centre of diphenyl NI **1**, the carbenoid C1 carbon, and the most electrophilic centre of carvone **3**, the *β*-conjugated C4 carbon (see Section S1 in Supplementary Material); (iii) the C−C and C−N distances associated to the formation of the corresponding single bonds, > 2.2 Å, indicating that formation of these single bonds has not started yet at any of the four TSs [21]; and (iv) a comparison of the geometrical parameters obtained at the B3LYP/6-31G(d) level, in the gas phase and in DCM, and those obtained at the B3LYP/6-311G(d,p) level in DCM, shows that there are no significant differences (see Figure 2).

Finally, in order to evaluate the polar nature of the 32CA reaction of diphenyl NI **2a** with (R)-carvone **1**, the global electron density transfer (GEDT) [34] at the more favourable regioisomeric TSs was analysed. Reactions with GEDT values of 0.0 e correspond to non-polar processes, while values above 0.2 e correspond to polar processes. The values of GEDT, which fluxes from the diphenyl NI framework to the (R)-carvone **1** at the more favourable regioisomeric TSs are 0.06 e at **TS-1-NI** and -0.02 e at **TS-3-NI**. These negligible values indicate that this 32CA reaction has non-polar character as a consequence of the moderate electrophilic character of (R)-carvone **1**, in clear agreement with the analysis of the CDFT indices.

#### 2.3.2. Study of the 32CA Reactions of Phenyl NO **4a** with (R)-Carvone **1**

**Scheme 3:**
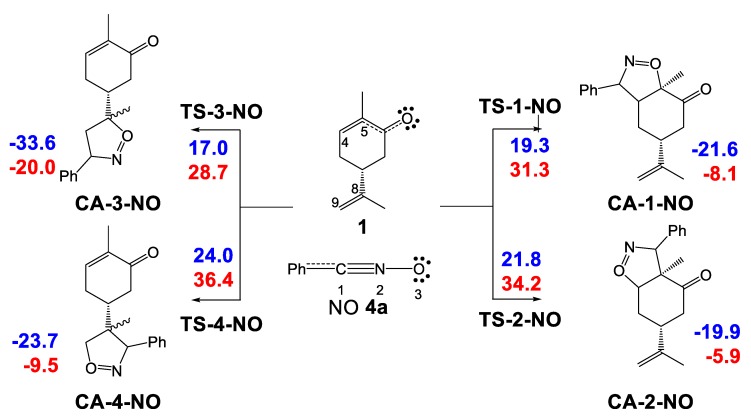
Selected reaction paths associated with the 32CA reaction of phenyl NO **4a** with (R)-carvone **1**. B3LYP/6-311G(d,p) relative enthalpies, in blue, and relative Gibbs free energies, in red, computed at 0 ˚C in DCM, are given in kcal·moL^−1^. The energy reference is the separated reagents.

Just as in the 32CA reaction of diphenyl NI **2a**, eight reactive paths are feasible for this 32CA reaction due to the non-symmetry of phenyl NO **4a** and (R)-carvone **1**. Four reaction paths were considered: the attack to the C4−C5 or C8−C9 double bonds of (R)-carvone **1**, and the regioisomeric approach modes of phenyl NO **4a** to these C−C double bonds (see Scheme 3). Facial selectivity was not considered [16]. Analysis of the stationary points involved in the four reaction paths indicates that this 32CA reaction takes place also through a one-step mechanism. Thus, four TSs and the corresponding isoxazoles were located and characterised (see Scheme 3). B3LYP/6-311G(d,p) relative enthalpies and Gibbs free energies in DCM are given in Scheme 3. The total and relative energies in the gas phase and in DCM and the thermodynamic data are given in Tables S5 and S7 in Supplementary Material.

The B3LYP/6-311G(d,p) activation enthalpies associated with the four competitive reaction paths are 19.3 (**TS-1-NO**) 21.8 (**TS-2-NO**), 17.0 (**TS-3-NO**), and 24.0 (**TS4-NO**) kcal·moL^−1^, the formation of the isoxazoles being exothermic by 25–30 kcal·moL^−1^. Some appealing conclusions can be obtained from these energy results: (i) this 32CA reaction presents an activation enthalpy via **TS-3-NO** of 17.0 kcal·moL^−1^. The activation energy associated with the 32CA between the simplest NO **9** and ethylene is 15.8 kcal·moL^−1^ (see Table S8 in Supplementary Material); (ii) this 32CA reaction is 4.6 kcal·moL^−1^ more unfavourable than that involving carbenoid diphenyl NI **2a**, in clear agreement with the *zw-type* reactivity of phenyl NO **4a**; (iii) this 32CA reaction is completely regioselective, as **TS-4-NO** is 7.0 kcal·moL^−1^ higher in enthalpy than **TS-3-NO**; (iv) this 32CA reaction is highly chemoselective, as **TS-1-NO** is 2.3 kcal·moL^−1^ higher in energy than **TS-3-NO**; (v) the high exothermic character of the four reaction paths makes them irreversible. Consequently, the formation of the most favourable isoxazole **CA-3-NO** is subject to kinetic control.

**Figure 3:**
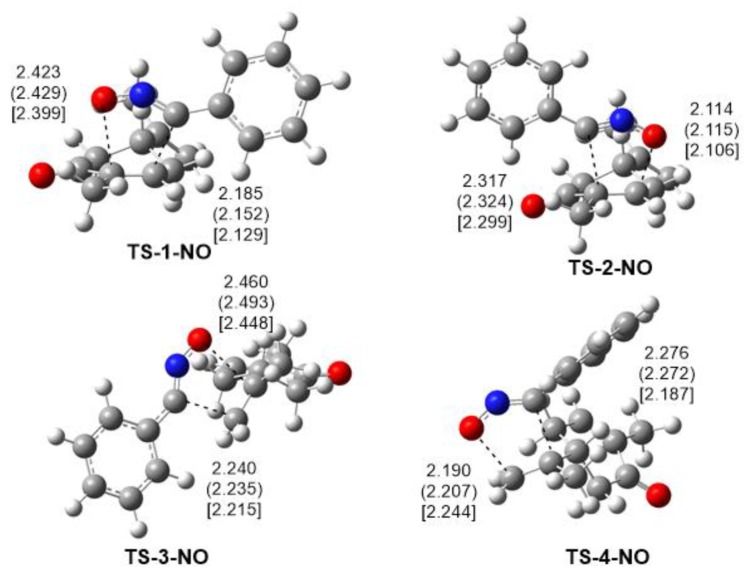
B3LYP/6-31G(d) optimised geometries, in gas phase and in DCM, of the TSs involved in the 32CA reaction of phenyl NO **4a** with (R)-carvone **1**. Distances are given in angstroms, Å. Values in DCM are given in parentheses, while the B3LYP/6-311G(d,p) values in DCM are given in brackets.

The geometries of the TSs in the gas phase and in DCM are given in Figure 3. The distances between the interacting C1 and Cx and O3 and Cy centres at the gas phase TSs are 2.185 (C1−C4) and 2.423 (O3−C5) Å at **TS-1-NO**, 2.114 (O3−C4) and 2.317 (C1−C5) Å at **TS-2-NO**, 2.240 (C1−C8) and 2.460 (O3−C9) Å at **TS-3-NO**, and 2.190 (O3−C8) and 2.276 (C1−C9) Å at **TS-4-NO**. Some appealing conclusions can be drawn from these geometrical parameters: (i) the more favourable regioisomeric TSs, **TS-1-NO,** and **TS-3-NO**, are more asynchronous than **TS-2-NO** and **TS-4-NO**; (ii) at the more favourable **TS-1-NO** and **TS-3-NO**, the C−C and C−O distances indicate that the formation of the C−C single bond is more advanced than the C−O one; (iii) similar to the 32CA reaction involving diphenyl NI **1**, the C−C and C−O distances associated to the formation of the corresponding bond, >2.1 Å, indicating that the formation of these single bonds has not started yet at any of the four TSs [21]; and (iv) a comparison of the geometrical parameters obtained at the B3LYP/6-31G(d) level, in the gas phase and in DCM, and those obtained at the B3LYP/6-311G(d,p) level in DCM, shows that there are no significant differences (see Figure 3).

The GEDT, which fluxes from the carvone framework to the NO one, at the more favourable regioisomeric TSs is 0.00e at **TS-1-NO** and 0.05e at **TS-3-NO**. These negligible values indicate that this 32CA reaction, just as that involving NI **2a**, has a non-polar character, in clear agreement with the analysis of the CDFT indices.

### 2.4. ELF Topological Analysis of the C−C and C−N Bond Formation along the Most Favourable Reaction Paths Associated with the 32CA Reaction of Diphenyl NI ***2a*** and Phenyl NO ***4a*** with (R)-Carvone ***1***

In order to characterise the formation of the two new C−C and C−N single bonds along the most favourable reaction paths associated with the 32CA reactions of diphenyl NI **2a** and phenyl NO **4a** with(R)-carvone **1**, a topological analysis of the ELF of the structures of the intrinsic reaction coordinate (IRC) paths directly involved in the single bond formation was performed. These structures were selected by applying the Bonding Evolution Theory (BET) [35] along the corresponding gas phase B3LYP/6-31G(d) IRCs. The thorough topological analysis of the ELF is given in Section 2 and Section 3 the Supplementary Material, while the most relevant conclusions arising from it are given below.

#### 2.4.1. ELF Topological Analysis of the C−C and C−N Bond Formation along the Most Favourable Reaction Path Associated with the 32CA Reaction of Diphenyl NI **2a** with (R)-Carvone **1**

**Figure 4:**
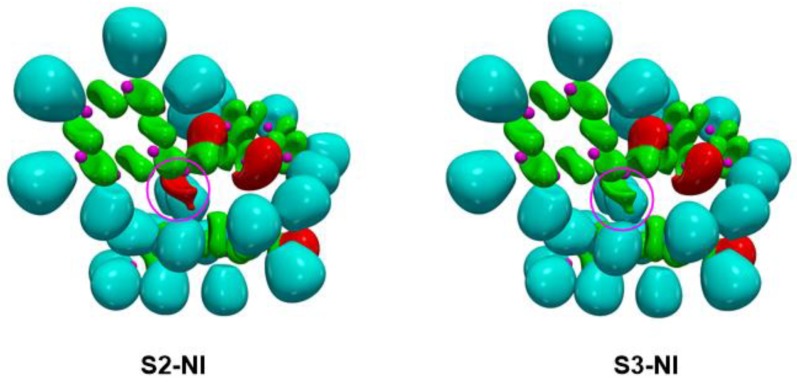
B3LYP/6-31G(d) ELF localisation domains, represented at an isosurface value of ELF = 0.75, of the structures **S2-NI** and **S3-NI** involved in the formation of the first C3−C4 single bond along the 32CA reaction between of diphenyl NI **2a** and (R)-carvone **1**.

**Figure 5:**
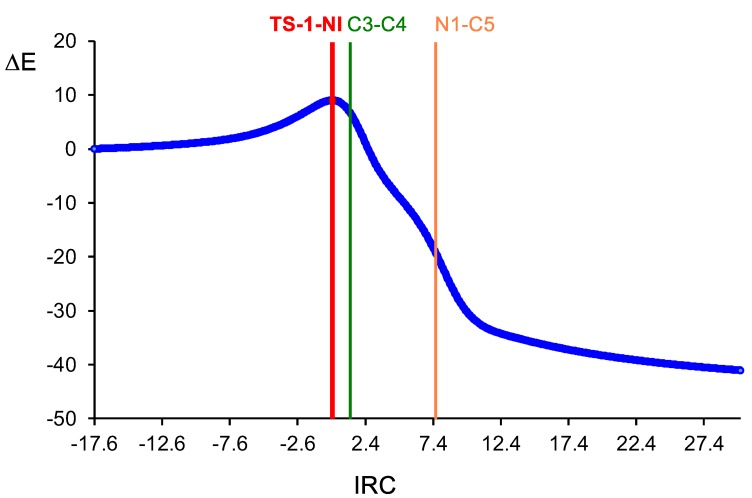
Gas-phase B3LYP/6-31G(d) IRC associated with the 32CA reaction between diphenyl NI **2a** and (R)-carvone **1**. The red line indicates the position of **TS-1-NI**, the green line indicates the position of the formation of the first C3−C4 single bond, and the orange line indicates the position of the formation of the second N1−C5 single bond. Relative energies (ΔE, in kcal·moL^−1^) are given with respect to the first structure of the reaction path, **S1-1-NI**.

Some appealing conclusions can be drawn from the topological analysis of the ELF along the 32CA reaction involving diphenyl NI **2a**: (i) the moderate activation enthalpy of the non-polar 32CA reaction between diphenyl NI **2a** and(R)-carvone **1**, 12.4 kcal·moL^−1^, can mainly be related to the depopulation of the N2−C3 bonding region of the diphenyl NI fragment, which leads to the formation of non-bonding electron density at the N2 nitrogen. Due to the non-polar character of the reaction, these bonding changes cannot be favoured [36]; (ii) formation of the first C3−C4 single bond takes place at **S3-NI**, at a C−C distance of ca. 1.98 Å and with an initial population of 1.95e, by donation, in an 80:20 ratio, of the non-bonding electron density of the C3 carbenoid centre of diphenyl NI **2a** to a C4 *pseudoradical* centre created from the depopulation of the conjugated C4−C5 bonding region of (R)-carvone **1** along the reaction path (see Figure 4) [24]; (iii) formation of the second N1−C5 single bond takes place at the end of the reaction path at **S5-NI**, at an N−C distance of ca. 1.85 Å and with an initial population of 1.13e, by donation of non-bonding electron density of the N1 nitrogen to the C5 carbon; (iv) despite the non-polar character of the reaction, the considerably high degree of asynchronicity, ca. 82.5%, computed as the separation between **S3-NI** and **S5-NI** relative to **TS-1-NI** in the IRC, suggests a *two-stage one-step* mechanism [37] (see Figure 5); (v) formation of the first C3−C4 single bond involves the most nucleophilic centre of diphenyl NI **2a**, which corresponds to the carbenoid C1 carbon, and the most electrophilic of (R)-carvone **1**, the *β*-conjugated position (see section S2 in Supplementary Material) [23]. This behaviour makes it possible to explain the chemo- and regioselectivity found experimentally in this 32CA reaction [16]; and finally, (vi) the present bond formation patterns for C−C and N−C single bonds agree with those previously reported in *cb-type* 32CA reactions [21].

#### 2.4.2. ELF Topological Analysis of the C−C and C−O Bond Formation along the Most Favourable Reaction Paths Associated with the 32CA Reaction of Phenyl NO **4a** with (R)-Carvone **1**

**Figure 6:**
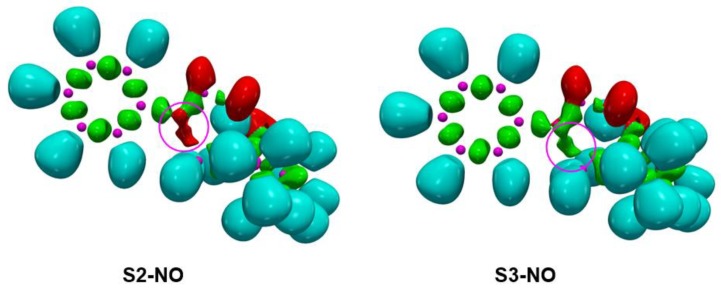
B3LYP/6-31G(d) ELF localisation domains, represented at an isosurface value of ELF = 0.75, of the structures **S2-NO** and **S3-NO** involved in the formation of the first C3−C4 single bond along the 32CA reaction between of phenyl NO **4a** and (R)-carvone **1**.

**Figure 7:**
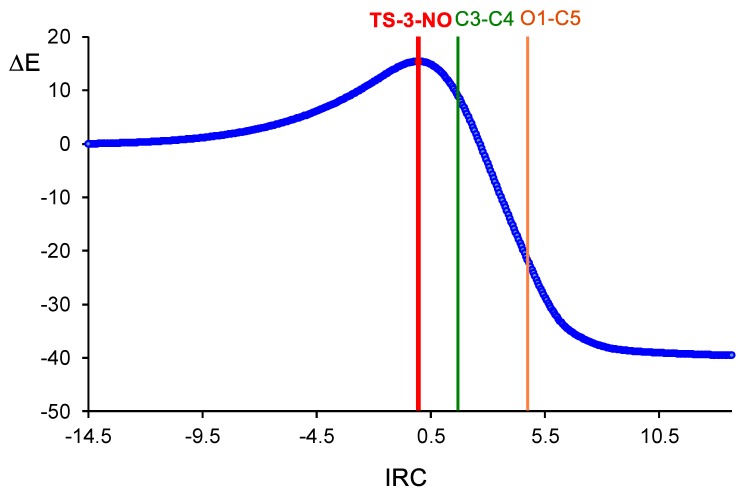
Gas-phase B3LYP/6-31G(d) IRC associated with the 32CA reaction between phenyl NO **4a** and (R)-carvone **1**. The red line indicates the position of **TS-3-NO**, the green line indicates the position of the formation of the first C3−C4 single bond, and the orange line indicates the position of the formation of the second O1−C5 single bond. Relative energies (ΔE, in kcal·moL^−1^) are given with respect to the first structure of the reaction path, **S1-NO**.

Some appealing conclusions can be drawn from the topological analysis of the ELF along the 32CA reaction involving phenyl NO **4a**: (i) the high activation enthalpy of the non-polar 32CA reaction between phenyl NO **4a** and the isopropenyl C−C double bond of (R)-carvone **1**, 17.0 kcal·moL^−1^, can mainly be related to the depopulation of the N2−C3 bonding region of the NO fragment, which leads to the formation of non-bonding electron density at the two N2 nitrogen and C3 carbon. Just as in the 32CA reaction involving diphenyl NI **2a**, the required bonding changes cannot be favoured due to the non-polar character of the reaction [36]; (ii) formation of the first C3−C4 single bond takes place at **S3-NO**, at a C−C distance of ca. 1.96 Å and with an initial population of 1.33e, by sharing part of the non-bonding electron density of the C3 carbon, 0.77 e, created from the depopulation of the N2−C3 bonding region of phenyl NO **2**, and that of the C4 *pseudoradical* centre, 0.53 e, created from the depopulation of the C4−C5 bonding region of (R)-carvone **1** in an approximate 60:40 ratio, respectively (see Figure 6) [22]; (iii) formation of the second O1−C5 single bond takes place at the end of the reaction path at **S5-NO**, at an O−C distance of ca. 1.80 Å and with an initial population of 0.71 e, by donation of non-bonding electron density of the O1 oxygen to the small amount of non-bonding electron density created at the C5 carbon in an approximate 77:33 ratio; (iv) this reaction proceeds with a relatively low degree of asynchronicity, ca. 63.5%, computed as the separation between **S5-NO** and **S3-NO** relative to **TS-3-NO** in the intrinsic reaction coordinate (IRC) (see Figure 7); and finally, (v) the present bond formation patterns for the C−C and O−C single bonds agree with those previously reported in non-polar *zw-type* 32CA reactions [21,22].

## 3. Conclusions

The 32CA reactions of diaryl NI **2a** and aryl NO **4a** with (R)-carvone **1**, recently studied experimentally by Ait Itto et al. [16], have been studied within the MEDT at the B3LYP/6-31G(d) and B3LYP/6-311G(d,p) computational levels, in the gas phase and in DCM.

The topological analysis of the ELF of diaryl NI **2a** and aryl NO **4a** allows characterising their electronic structure as carbenoid and zwitterionic TACs, respectively. Analysis of the CDFT indices indicates that the corresponding 32CA will have a low polar character given the low electrophilic character of (R)-carvone **1**.

Four competitive reaction paths have been considered; two pairs of regio- and two pairs of chemoisomeric reaction paths. These 32CA reactions take place through a one-step mechanism. The activation enthalpies associated with these 32CA reactions are 12.4 kcal·moL^−1^ (**TS-1-NI**) and 17.0 kcal·moL^−1^ (**TS-3-NO**). As expected, the *zw-type* 32CA reaction involving phenyl NO **4a** presents a higher activation enthalpy than that associated to the *cb-type* 32CA reaction involving diphenyl NI **2a** [21]. The two 32CA reactions are regioselective. However, while the 32CA reaction of phenyl NO **4a** is highly chemoselective, preferring the attack on the isopropenyl C−C bond of (R)-carvone **1**, that of diphenyl NI **2a** presents low chemoselectivity preferring the attack to the *α*,*β*-conjugated C−C bond of **1**. Although MPWB1K and M06-2X functionals correctly predict the total chemoselectivity for the 32CA reaction of diphenyl NI **2a**, these functionals predict low chemoselectivity for the reaction with phenyl NO **4a**. In any case, four functionals predict a change of reactivity of carbenoid diphenyl NI **2a** with respect to that of zwitterionic, in complete agreement with the experimental outcomes.

ELF analysis of the formation of the two new C−C and C−N single bonds indicates that the *cb-type* 32CA reaction of diphenyl NI **2a** begins by the C−C bond formation between the carbenoid C1 carbon of NI **2a** and the *β-*conjugated position of (R)-carvone **1** by donation [23]. On the other hand, the *zw-type* 32CA reaction of zwitterionic phenyl NO **4a** begins by the C−C bond formation between the C1 carbon of **4a** and the methylene C4 carbon of the isopropenyl framework of (R)-carvone **1**, by sharing the electron density of the C1 and C4 *pseudoradical* centres created along the reaction path [22].

The present MEDT study, based on two experimental 32CA reactions [16], supports the recent classification of the 32CA reactions made by Domingo and Ríos-Gutiérrez in *pdr-type*, *pmr-type*, *cb-type* and *zw-type* reactions, and asserts MEDT, based on the analysis of molecular electron density, as an interpretative chemical theory able to explain chemical reactivity in Organic Chemistry.

## 4. Computational Methods

All stationary points were optimised using the B3LYP functional [38,39] together with the 6-31G(d) and 6-311G(d,p) basis sets [40]. As this functional did not reproduce the total chemoselectivity found in the 32CA reaction of diphenyl NI **2a**, the MPWB1K [41], M06-2X [42], B3LYP-D3 [43], and wB97XD [44] functionals were tested (see Supplementary Material). Although these functionals reproduced the experimental chemoselectivity of diphenyl NI **2a**, all of them failed to predict the chemoselectivity found in the 32CA reaction of diphenyl phenyl NO **4a**. The optimisations were carried out using the Berny analytical gradient optimisation method [45,46]. The stationary points were characterised by frequency computations in order to verify that TSs have one and only one imaginary frequency. The gas-phase B3LYP/6-31G(d) IRC paths [47] were traced in order to check and obtain the energy profiles connecting each TS to the two associated minima of the proposed mechanism, i.e., reactants and products, using the second-order González–Schlegel integration method [48,49]. Solvent effects of DCM were taken into account by full optimisation of the gas phase stationary points using the polarisable continuum model (PCM) [50,51] in the framework of the self-consistent reaction field (SCRF) [52,53,54]. Values of B3LYP/6-311G(d,p) enthalpies, entropies, and Gibbs free energies in DCM were calculated with standard statistical thermodynamics at 273.15 K and 293.15 K and 1 atm [40].

The electronic structures of the stationary points were characterised by NPA [55,56], and by the topological analysis of the ELF [25]. CDFT reactivity indices [28,29] were computed at the B3LYP/6-31G(d) level using the equations given in reference 29. GEDT [34] was computed by the sum of the atomic charges (q) of the atoms belonging to each framework at the TSs; GEDT = Σq_f_.

All computations were carried out with the Gaussian 16 suite of programs [57]. ELF studies were performed with the TopMod program [58], using the corresponding B3LYP/6-31G(d) monodeterminantal wavefunctions and considering the standard cubical grid of step size of 0.1 Bohr. The molecular geometries and ELF basin attractor positions were visualised using the GaussView program [59], while the ELF localisation domains were represented by means of the Paraview software at an isovalue of 0.75 a.u. [60,61].

## Figures and Tables

**Table 1:** B3LYP/6-31G(d) global electronic chemical potential, μ, chemical hardness, η, electrophilicity, ω, and nucleophilicity, *N*, in eV, of NIs **2a**, **8**, NOs **4a, 9** and (R)-carvone **1**.

	μ	η	ω	*N*
diphenyl NI **2a**	−3.36	3.78	1.50	3.87
phenyl NO **4a**	−3.83	5.02	1.46	2.78
(R)-carvone **1**	−3.84	5.23	1.41	2.67
simplest NI **8**	−3.55	5.87	1.07	2.64
simplest NO **9**	−3.40	7.94	0.73	1.75

## Supplementary Materials

The following are available online. Comparative analysis of the relative energies obtained using the standard B3LYP, MPWB1K, M06-2X, B3LYP-D3, and wB97XD functionals. ELF topological analysis of the C−C and C−N bond formation along the 32CA reactions of diphenyl NI **2a** and phenyl NO **4a** with (R)-carvone **1**. Tables with the B3LYP/6-31G(d) total and relative energies, in the gas phase and in DCM, of the stationary points associated with the 32CA reactions of diphenyl NI **2a** and phenyl NO **4a** with (R)-carvone **1**, as well as with the B3LYP/6-311G(d,p) thermodynamic data in DCM. Tables with the B3LYP/6-311G(d,p) thermodynamic data in DCM of the stationary points associated to the 32CA reaction of simplest NI **8** and simplest NO **9** with ethylene **6**.
