# Supplementary material for: Unveiling the Different Chemical Reactivity of Diphenyl Nitrilimine and Phenyl Nitrile Oxide in [3+2] Cycloaddition Reactions with (R)-Carvone through the Molecular Electron Density Theory
Source: Molecules. 2020 Feb 28;25(5):1085. doi: 10.3390/molecules25051085 (PMC7179222; doi:10.3390/molecules25051085)
Supplement: Supplementary file 1 [file molecules-25-01085-s001.pdf]

Supplementary Material

Unveiling the Different Chemical Reactivity of DiphenylNitrilimine and Phenyl Nitrile Oxidein [3+2] Cycloaddition Reactions with (R)-Carvone through the Molecular Electron Density Theory

Mar Ríos-Gutierrez,[1,2]* Luis R. Domingo,[1] M'hamedEsseffar,[3] Ali Oubella,[3] My Youssef Ait Itto[3]

[1] Department of Organic Chemistry, University of Valencia, Dr.Moliner 50, Burjassot, E-46100 Valencia, Spain

[2] Department of Chemistry and Chemical Biology, McMaster University, 1280 Main Street West, Hamilton, Ontario L8S 4L8, Canada

[3] Laboratoire de Synthese Organique et Physico-Chimie Moléculaire, Département de Chimie, Faculté des Sciences, Semlalia, B.P 2390, Marrakech, 40001, Morocco

Index

S2 Comparative analysis of the relative energies obtained using the B3LYP, MPWB1K, M06-2X, B3LYP-D3 and wB97XD functionals.

S4 Analysis of the nucleophilic Parr functions of diphenyl NI **2a** and the electrophilic Parr functions of (R)-carvone **1**

S5 ELF topological analysis of the C–C and C–N bond formation along the 32CA reaction between diphenyl NI **2a** and (R)-carvone **1**.

S8 ELF topological analysis of the C–C and C–N bond formation along the 32CA reaction between phenyl NO **4a** and (R)-carvone **1**.

S12 References.

S14 Tables with the B3LYP/6-31G(d) total and relative energies, in gas phase and in DCM, of the stationary points associated to the 32CA reactions of diphenyl NI **2a** and phenyl NO **4a** with (R)-carvone **1**.

S15 Tables with the B3LYP/6-311G(d,p) thermodynamic data in DCM of the stationary points associated to the 32CA reaction ofdiphenyl NI **2a** and phenyl NO **4a** with (R)-carvone **1**.

S16 Tables with the B3LYP/6-311G(d,p) thermodynamic data in DCM of the stationary points associated to the 32CA reaction ofsimplest NI **8** and simplest NO **9** with ethylene **6**.

1. Comparative analysis of the relative energies obtained using the B3LYP, MPWB1K, M06-2X, B3LYP-D3 and WB97XD functionals.

32CA reactions have been widely computationally studied using different DFT functionals, the B3LYP [1,2], MPWB1K [3] and M06-2X [4] being the most used. A comparative analysis of the relative energies in DCM of the stationary points associated with the two more favourable chemoselective reaction paths associated to the 32CA reactions of (R)-carvone **1** with diphenyl NI **2a** and with phenyl NO **4a** obtained using these functionals was performed. In addition, the B3LYP-D3 [5] and wB97XD [6] functionals, which are capable of capturing also long-range interactions, were also tested. The total and relative energies are given in Table S1.

A comparative analysis of the relative energies obtained with the five functionals shows that the activation energies decrease in the order B3LYP>MPWB1K>M06-2X> wB97XD> B3LYP-D3, while the exothermic character of these reaction increases in the order B3LYP<B3LYP-D3>MPWB1K>M06-2X<wB97XD. A similar trend was observed in Diels-Alder reactions for the functionals B3LYP, MPWB1K and M06-2X [7]. Unlike the B3LYP functional, the MPWB1K, M06-2X, B3LYP-D3 and wB97XD functionals suggest a total chemoselectivity for the 32CA reaction of (R)-carvone **1** with diphenyl NI **2a**, but for the 32CA reaction with phenyl NO **4a**, the MPWB1K functional reduces the chemoselectivity, while the M06-2X and B3LYP-D3 functionals predicts the reaction to be non-chemoselective. It is interesting to note that the wB97XD functional even predicts an inverse chemoselectivity to that experimentally observed.

Consequently, none of the five functionals is able to predict completely the experimental outcomes for the two 32CA reactions. This finding is a consequence of the different nature of the TSs of the two 32CA reactions, a *cb-type* and a *zw-type*, which are differently considered for these functional. This comparative analysis also emphasized that the M06-2X, B3LYP-D3 and wB97XD functionals yield very low activation energies because they significantly underestimate the energies of the TSs [8].

Table S1. B3LYP/6-311G(d,p), MPWB1K/6-311G(d,p), M06-2X/6-311G(d,p), B3LYP-D3/6-311G(d,p) and wB97XD/6-311G(d,p) total, E in au, and relative energies, ΔE in kcal·mol^{-1}, in DCM, of the stationary points associated to the more favourable chemoselective reaction paths of the 32CA reactions of diphenyl NI **2a** and phenyl NO **4a** with (R)-carvone **1**.

	B3LYP		MPWB1K		M06-2X	
	E	ΔE	E	ΔE	E	ΔE
NI **2a**	-610.978864		-610.650088		-610.713541	
(R)-carvone **1**	-464.824178		-464.562650		-464.610215	
**TS-1-NI**	-1075.784023	11.9	-1075.201234	7.2	-1075.322131	1.0
**TS-3-NI**	-1075.783388	12.3	-1075.196473	10.2	-1075.315489	5.2
**CA-1-NI**	-1075.861024	-36.4	-1075.306394	-58.8	-1075.419000	-59.8
**CA-3-NI**	-1075.873967	-44.5	-1075.315759	-64.6	-1075.427116	-64.9
NO **4a**	-399.747470		-399.537414		-399.574296	
**TS-1-NO**	-864.541204	19.1	-864.071505	17.9	-864.163494	13.2
**TS-3-NO**	-864.544899	16.8	-864.073300	16.8	-864.163693	13.1
**CA-1-NO**	-864.610013	-24.1	-864.165587	-41.1	-864.252954	-42.9
**CA-3-NO**	-864.629392	-36.2	-864.181722	-51.2	-864.267465	-52.1

	B3LYP-D3		wB97XD	
	E	ΔE	E	ΔE
NI **2a**	-610.995171		-610.744747	
(R)-carvone **1**	-464.845961		-464.671466	
**TS-1-NI**	-1075.844819	-2.3	-1075.417846	-1.0
**TS-3-NI**	-1075.838163	1.9	-1075.410910	3.3
**CA-1-NI**	-1075.920321	-49.7	-1075.515747	-62.5
**CA-3-NI**	-1075.929933	-55.7	-1075.525096	-68.3
NO **4a**	-399.754861		-399.595318	
**TS-1-NO**	-864.585386	9.7	-864.247565	12.1
**TS-3-NO**	-864.586055	9.3	-864.246874	12.5
**CA-1-NO**	-864.653425	-33.0	-864.348373	-51.2
**CA-3-NO**	-864.669591	-43.2	-864.333447	-41.8

*2. Analysis of the nucleophilic P_k^- Parr functions of diphenyl NI **2a** and the electrophilic P_k^+ Parr functions of (R)-carvone **1***

In order to characterise the most nucleophilic and electrophilic centers of diphenyl NI **2a** and (R)-carvone **1**, the nucleophilic P_k^- Parr functions of diphenyl NI **2a** and the electrophilic P_k^+ Parr functions of (R)-carvone **1** were analysed (see Figure S1) [9].

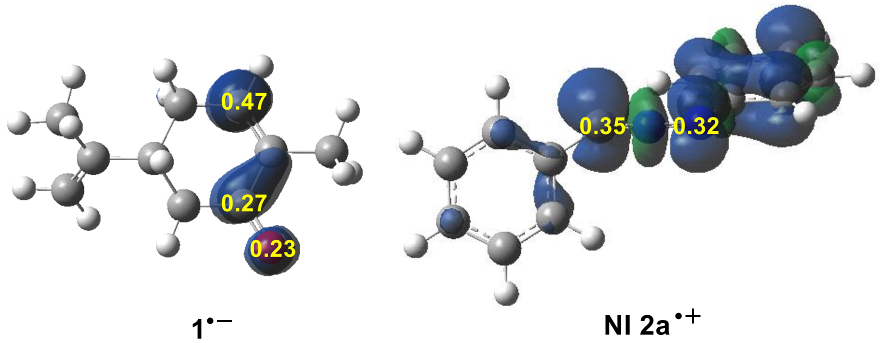

Figure S1. 3D representations of the Mulliken ASD of the radical anion **1**$^{\cdot-}$ and the radical cation diphenyl NI **2a**$^{\cdot+}$, together with the electrophilic P_k^+ Parr functions of (R)-carvone **1** and the nucleophilic P_k^- Parr functions of diphenyl NI **2a**, obtained at the B3LYP/6-31G(d) level.

The electrophilic P_k^+ Parr functions at (R)-carvone **1** show that the β-conjugated C4 carbon, P_k^+(C4) = 0.47 is clearly the most electrophilic center of this molecule. Note that the C4 carbon is twice electrophilically activated than the carbonyl C5 carbon. Consequently, it is expected that nucleophiles attack to C4 carbon of (R)-carvone**1**. Note that the C8−C9 double bond of carbon does not present any electrophilic activation, thus, no participating in polar reactions.

Conversely, analysis of the nucleophilic P_k^- Parr functions at the reactive sites of diphenyl NI **2a** indicates that both terminal N1 and C3 nuclei are strongly nucleophilically activated to a similar extent, P_k^-(C1) = 0.35 and P_k^-(N3) = 0.32, the carbenoid C1 carbon having a slightly more nucleophilic character (see Figure S1). Note that the more nucleophilically activated C3 carbon corresponds to the less electronegative atom.

*3. ELF topological analysis of the C–C and C–N bond formation along the 32CA reaction between diphenyl NI **2a** and (R)-carvone **1**.*

In order to characterise the C–C and C–N bond formation along the 32CA reaction between diphenyl NI **2a** and (R)-carvone **1**, a topological analysis of the Electron Localisation Function (ELF) [10] of the structures of the IRC directly involved in the formation of the new C–C and C−O single bonds was performed. These structures were selected by applying the Bonding Evolution Theory (BET) [11] along the IRC associated with the most favourable reaction path. The populations of the most relevant ELF valence basins, the C–C and C–O forming bond distances, global electron density transfer (GEDT) [12] and relative energies of the selected structures of the IRC are gathered in Table S2.

The electronic structure of **S1-NI**, d(N1−C5) = 3.364 Å and d(C3−C4) = 3.541 Å, which is the first structure of the reaction path, resembles that of the separated reagents. Thus, at the NI framework, two V(N1) and V(C3) monosynaptic basins, integrating 3.33 e and 1.43 e, are observed, which can be associated with less than two N1 nitrogen lone pairs and a C3 carbenoid center, respectively, while the other V(N1,N2), V(N2,C3) and V’(N2,C3) disynaptic basins can be associated to an N1−N2 single bond and an overpopulated N2−C3 double bond with total populations of 2.09 e and 4.79 e. The two V(C4,C5) and V’(C4,C5) disynaptic basins integrating a total population of 3.47 e characterize the C4−C5 double bond of the (R)-carvone moiety.

At **TS-1-NI**, d(N1−C5) = 2.642 Å and d(C3−C4) = 2.213 Å, a new V(N2) monosynaptic basin integrating 2.15 e has appeared mainly as a consequence of the strong depopulation of the two V(N2,C3) and V’(N2,C3) disynaptic basins by 1.85 e to 2.94 e, but also due to the depopulation of the V(N1,N2) disynaptic basin by 0.22 e to 1.87 e. This V(N2) monosynaptic basin can be related to the non-bonding electron density present at the N2 nitrogen of product **CA-1-NI**. Interestingly, despite the significant depopulation of the N2−C3 bonding region, the population of the V(C3) monosynaptic basin has increased by only 0.07 e. Another topological change is the merger of the two V(C4,C5) and V’(C4,C5) disynaptic basins present at **S1-NI** into a single V(C4,C5) disynaptic basin integrating 3.23 e, as a consequence of their depopulation by a total of 0.24 e.

At **S2-NI**, d(N1−C5) = 2.556 Å and d(C3−C4) = 1.990 Å, which is the structure before the formation of the first single bond, the most relevant topological change with

respect to the previous structure is the presence of a new V(C4) monosynaptic basin integrating 0.39 e, which clearly comes from the depopulation of the V(C4,C5) disynaptic basin by 0.52 e to 2.71 e. The population of the V(N2) monosynaptic basin keeps growing to 2.38 e, while that of the V(N1) monosynaptic basin has decreased to 3.10 e.

At **S3-NI**, d(N1–C5) = 2.552 Å and d(C3–C4) = 1.979 Å, the two V(C3) and V(C4) monosynaptic basins present at **S7'** merge into a new V(C3,C4) disynaptic basin integrating an initial population of 1.95 e. The population of this V(C3,C4) disynaptic basin, which can be related to formation of the new C3–C4 single bond, comes from an 80(C3):20(C4) contribution; consequently, formation of the first C3–C4 single bond takes place at a C–C distance of ca. 1.98 Å by donation of the non-bonding electron density of the C3 carbenoid center to the C4 *pseudoradical* center.

At **S4-NI**, d(N1–C5) = 1.857 Å and d(C3–C4) = 1.546 Å, which is the structure before the formation of the second N1–C5 single bond, only variations in the ELF valence basin populations are noticed. While the population of the V(N1,N2) disynaptic basin has decreased to 1.59 e, the populations of the V(N1), V(N2) and V(N2,C3) basins have increased to 3.28 e, 2.75 e and 3.13e, respectively. Note that after a slight depopulation along the reaction path, the V(N1) monosynaptic basin recovers almost its initial population of **S1-NI**(see Table S2). The most drastic depopulation is that experienced by the V(C4,C5) disynaptic basin, which reaches 2.06 e after losing 0.63 e.

At **S5-NI**, d(N1–C5) = 1.848 Å and d(C3–C4) = 1.545 Å, together with the depopulation of the V(N1) monosynaptic basin by 1.11 e to 2.17 e, a new V(N1,C5) disynaptic basin is created with an initial population of 1.13 e. This topological change indicates that formation of the second N1–C5 single bond takes place at an N–C of ca. 1.85 Å by donation of non-bonding electron density of the N1 nitrogen to the C5 carbon.

Finally, at product **CA-1-NI**, d(N1–C5) = 1.491 Å and d(C3–C4) = 1.525 Å, the most remarkable topological change is the split of the V(N1) monosynaptic basin, whose total population has increased by 0.28 e, into two V(N1) and V'(N1) monosynaptic basins integrating 1.15 e and 1.30 e as a consequence of the planar arrangement of the N1 nitrogen. The population of the V(N1,C5) disynaptic basin reaches 0.44e, while the V(N1,N2) and V(C4,C5) disynaptic basins are still depopulated to 1.43e and 1.89e. The other V(N2), V(N2,C3) and V(C3,C4) basins end up with 2.85e, 3.07e and 2.02e, respectively.

Table S2. ELF valence basin populations, distances of the forming bonds, relative[a] electronic energies, and GEDT of **TS-1-NI** and the selected structures of the IRC involved in the formation of the new N1[C3]–C5[C4] single bonds along the 32CA reaction of diphenyl NI **2a** with (R)-carvone **1**, obtained in gas phase at the B3LYP/6-31g(d) level. The electron populations and GEDT values are given in average number of electrons, e, distances in angstroms, Å, and relative energies in kcal·mol^{-1}.

Structures	**S1-NI**	**TS-1-NI**	**S2-NI**	**S3-NI**	**S4-NI**	**S5-NI**	**CA-I-NI**
d(N1–C5)	3.364	2.642	2.556	2.552	1.857	1.848	1.491
d(C3–C4)	3.541	2.213	1.990	1.979	1.546	1.545	1.525
ΔE	0.0	9.0	6.9	6.7	−18.9	−19.2	−41.1
GEDT	0.01	0.06	0.05	0.05	-0.11	−0.12	−0.18
V(N1,N2)	2.09	1.87	1.83	1.83	1.59	1.58	1.43
V(N2)		2.15	2.38	2.38	2.75	2.76	2.85
V(N2,C3)	2.60	2.94	2.83	2.83	3.13	3.13	3.07
V'(N2,C3)	2.19						
V(C4,C5)	1.73	3.23	2.71	2.69	2.06	2.06	1.89
V'(C4,C5)	1.74						
V(N1)	3.33	3.16	3.10	3.10	3.28	2.17	1.15
V'(N1)							1.30
V(C3)	1.43	1.50	1.55				
V(C4)			0.39				
V(C5)							
V(C3,C4)				1.95	1.96	1.96	2.02
V(N1,C5)						1.13	1.57

[a] Relative to the first structure of the IRC path, **S1-NI**.

*4. ELF topological analysis of the C–C and C–N bond formation along the 32CA reaction between phenyl NO **4a** and (R)-carvone **1**.*

In order to characterise the C–C and C–N bond formation along the 32CA reaction between phenyl NO **4a** and(R)-carvone **1**, a topological analysis of the ELF of the structures of the IRC directly involved in the formation of the new C–C and C–O single bonds was performed. These structures were selected by applying the BET along the IRC associated with the most favourable reaction path, involving the exocyclic ethylene framework. The populations of the most relevant ELF valence basins, the C–C and C–O forming bond distances, GEDT and relative energies of the selected structures of the IRC are gathered in Table S3.

At **S1-NO**, d(O1-C5) = 3.382 Å and d(C3-C4) = 3.938 Å, the ELF picture resembles that of the separated reagents. Thus, at the NO framework, the presence of two V(O1) and V'(O1) monosynaptic basins, integrating a total of 5.72 e, a V(O1,N2) integrating 1.55 e, together with two V(N2,C3) and V'(N2,C3) disynaptic basins integrating a total of 6.02 e, suggest a zwitterionic structure[13] with an N2–C3 triple bond, a depopulated O1–N2 single bond and O1 oxygen non-bonding electron density. On the other hand, the ethylene moiety is characterized by two V(C4,C5) and V'(C4,C5) disynaptic basins integrating a total of 3.59 e, which can be related to a C4–C5 double bond.

At **TS3-NO**, d(O1-C5) = 2.429 Å and d(C3-C4) = 2.242 Å, several ELF topological changes are observed. A new V(N2) monosynaptic basin, integrating 2.12e, has appeared while the two contiguous V(N2,C3) and V'(N2,C3) disynaptic basins have been strongly depopulated by a total of 2.94 e to 3.08 e. This depopulation can be associated to the initial rupture of the N2–C3 triple bond of the TAC framework. This fact, together with the depopulation of the C3–C3' bonding region to 2.25 e, may has also contributed to the creation of three V(C3), V'(C3) and V''(C3) monosynaptic basins integrating a total population of 1.37 e, which could be related to an underpopulated C3 carbenoid center. On the other hand, the V(C4,C5) disynaptic basin has been depopulated to 3.29 e, which could also be associated to the rupture of the isopropenylC4–C5 double bond of (R)-carvone**1**.

At **S2-NO**, d(O1–C5) = 2.242 Å and d(C3–C4) = 1.966 Å, which is the nearest structure associated with the formation to the first C3–C4 single bond, a new V(C4) monosynaptic basin is observed with a population of 0.53 e, probably coming from the

depopulation of the V(C4,C5) disynaptic basin by 0.65e to 2.64 e. This V(C4) monosynaptic basin can be associated with the C4 *pseudoradical* center demanded for the formation of C3–C4 single bonds [12]. Note that at **S2-NO**, the non-bonding electron density at the C3 carbon has increased to 1.54e as the N2–C3 and C3–C3' bonding regions keep depopulating to 2.85 e [V(N2,C3) and V'(N2,C3)] and 2.21 e [V(C3,C3')].

At **S3-NO**, d(O1–C5) = 2.234 Å and d(C3–C4) = 1.955 Å, together with the disappearance of the two V'(C3) and V''(C3) monosynaptic basins integrating 0.38 e and 0.77 e, as well as the V(C4) monosynaptic basin present at **S2-NO** with 0.53e, a new V(C3,C4) disynaptic basin has been created integrating an initial population of 1.33 e. This relevant ELF topological change indicates that formation of the first C3–C4 single bond takes place at a C–C distance of ca. 1.97 e by sharing part of the C3 carbon non-bonding electron density (0.77 e) and that of the C4 *pseudoradical* center. Note that after this process, a population of 0.39 e remains at the C3 carbon, while the total population of the V(N2,C3) and V'(N2,C3) disynaptic basins has increased by 0.36 e to 3.21 e, suggesting that the population of the V'(C3) monosynaptic basin has been redistributed into the N2–C3 bonding region.

At **S4-NO**, d(O1–C5) = 1.808 Å and d(C3–C4) = 1.595 Å, the strong depopulation of the V(C4,C5) and V'(C4,C5) disynaptic basins by a total of 0.55 e to 2.08 e have led to the creation of a new V(C5) monosynaptic basin, integrating 0.15 e at **S3-NO**, besides the increase of the population of the V(C3,C4) disynaptic basin associated to the new C3–C4 single bond to 1.87 e. Thus, at this structure, the C4–C5 bonding region could be already considered a single bond. Interestingly, while the total population of the two V(O1) and V'(O1) monosynaptic basins had somewhat decreased along the reaction path, at **S4-NO** it has increased by 0.24 e to 5.93 e and, additionally, a third V''(O1) monosynaptic basin has been created with a population of 0.47 e. These changes can be attributed to the depopulation of the V(O1,N2) disynaptic basin to 1.11 e. In addition, although the total population of the two V(N2,C3) and V'(N2,C3) disynaptic basins has increased by a total of only 0.06 e, these basins have merged into one single V(N2,C3) disynaptic basin integrating 3.27 e, while the V(N2) monosynaptic basin has reached 2.79e. Note, also, that the V(C3) monosynaptic basin has disappeared.

At **S5-NO**, d(O1–C5) = 1.796 Å and d(C3–C4) = 1.592 Å, a new V(O1,C5) disynaptic basin, integrating an initial population of 0.75 e, has been created as a consequence of the merger of the two V''(O1) and V(C5) monosynaptic basins present

at **S4-NO**. Consequently, formation of the second O1–C5 single bond takes place at an O–C distance of ca. 1.80 Å, through donation of part of the non-bonding electron density of the O1 oxygen (0.57 e) to the C5 *pseudoradical* center created along the reaction path. Note that the total population of the two remaining V(O1) and V'(O1) monosynaptic basins has decreased by 0.57 e to 5.36 e.

Finally, at **CA-3-NO**, d(O1–C5) = 1.477 Å and d(C3–C4) = 1.513 Å, only slight variations in the ELF valence basin populations, related to the energy and geometry relaxation of the molecular system, are noticed. The most marked change is the increase of the population of the recently formed V(O1,C5) disynaptic basin by 0.55e, reaching 1.26e while the V(C3,C4) disynaptic basin acquires 1.98e. These values clearly emphasize the strong polarization of the O1-C5 bonding region towards the O1 oxygen, whose associated population decreased to 5.00e [V(O1) and V'(O1)]. In the O1-N2-C3 framework, the changes are not significant, but the low population of the V(O1,N2) disynaptic basin, 0.98e, is noteworthy as it also suggests a strong polarization of the O1-N2 bonding region.

Table S3. ELF valence basin populations, distances of the forming bonds, relative[a] electronic energies, and GEDT of **TS-3-NO** and the selected structures of the IRC involved in the formation of the new O1[C3]–C5[4] single bonds along the 32CA reaction of phenyl NO **4a** with (R)-carvone **1**, obtained in gas phase at the B3LYP/6-31g(d) level. The electron populations and GEDT values are given in average number of electrons, e, distances in angstroms, Å, and relative energies in kcal·mol^{-1}.

Structures	**S1-NO**	**TS-3-NO**	**S2-NO**	**S3-NO**	**S4-NO**	**S5-NO**	**CA-3-NO**
d(O1–C5)	3.382	2.429	2.242	2.234	1.808	1.796	1.477
d(C3–C4)	3.938	2.242	1.966	1.955	1.595	1.592	1.513
ΔE	0.0	15.4	9.4	8.9	−21.0	−21.7	−39.6
GEDT	0.01	−0.04	−0.15	−0.15	−0.26	−0.27	−0.29
V(O1,N2)	1.55	1.40	1.31	1.30	1.11	1.10	0.98
V(N2)		2.12	2.46	2.47	2.79	2.80	2.92
V(N2,C3)	3.47	1.54	1.42	1.79	3.27	3.27	3.16
V'(N2,C3)	2.55	1.54	1.43	1.42			
V"(N2,C3)							
V(C3,C3')	2.53	2.25	2.21	2.22	2.23	2.23	2.26
V(C4,C5)	1.79	3.29	2.64	2.63	2.08	2.06	1.96
V'(C4,C5)	1.80						
V(O1)	3.35	2.88	2.88	2.89	2.76	2.71	2.44
V'(O1)	2.37	2.79	2.79	2.80	2.70	2.65	2.56
V"(O1)					0.47		
V(C3)		0.44	0.77	0.39			
V'(C3)		0.47	0.39				
V"(C3)		0.46	0.38				
V(C4)			0.53				
V(C3,C4)				1.33	1.87	1.87	1.98
V(C5)					0.14		
V(O1,C5)						0.71	1.26

[a] Relative to the first structure of the IRC path, **S1-NO**.

References

1. Becke, A.D. Density-functional thermochemistry. The role of exact Exchange. *J. Chem. Phys.* **1993**, *98*, 5648–5652.
2. Lee, C.; Yang, W.; Parr, R.G. Development of the Colle-Salvetti correlation-energy formula into a functional of the electron density. *Phys. Rev. B* **1988**, *37*, 785–789.
3. Zhao, Y.; Truhlar, D.G. Hybrid meta density functional theory methods for thermochemistry, thermochemical kinetics, and noncovalent Interactions: The MPW1B95 and MPWB1K models and comparative assessments for hydrogen bonding and van der Waals interactions. *J. Phys. Chem. A,* **2004**, *108*, 6908-6918.
4. Zhao, Y.; Truhlar, D.G. The M06 suite of density functionals for main group thermochemistry, thermochemical kinectics, noncovalent interactions, excited states, and transition elements: Two new functionals and systematic testing of four M06-class functionals and 12 other functionals. *Theor. Chem. Acc.* **2008**, *120*, 215–241.
5. Chai, J.-D.; Head-Gordon, M. Systematic optimization of long-range corrected hybrid density functionals. *Chem. Phys.* **2008**, *128*, 084106.
6. Grimme, S.; Antony, J.; Ehrlich, S.; Krieg, H. A consistent and accurate ab initio parameterization of density functional dispersion correction (DFT-D) for the 94 elements H-Pu, *J. Chem. Phys*. **2010**, *132*. 154104.
7. Domingo, L.R.; Ríos-Gutiérrez, M.; Pérez, P. How does the global electron density transfer diminish activation energies in polar cycloaddition reactions? A Molecular Electron Density Theory study *Tetrahedron* **2017**, *73*, 1718-1724.
8. Sobhi, C.; Nacereddine, A. K.; Djerourou, A.; Aurell, M.J.; Domingo, L.R. The role of the trifluoromethyl group in reactivity and selectivity in polar cycloaddition reactions. A DFT study. *Tetrahedron* **2012**, *68*, 8457-8462.

9 Domingo, L.R.; Pérez, P.; Sáez, J.A. Understanding the local reactivity in polar organic reactions through electrophilic and nucleophilic Parr functions *RSC Adv.* **2013**, *5*, 1486-1494.

10. Becke, A.D.; Edgecombe, K.E. A simple measure of electron localization in atomic and molecular-systems. *J. Chem. Phys.* **1990**, *92*, 5397-5403.

11 Krokidis, X.; Noury, S.; Silvi, B. Characterization of Elementary Chemical Processes by Catastrophe Theory. *J. Phys. Chem. A* **1997**, *101*, 7277-7282.

12. Domingo, L.R. A new C-C bond formation model based on the quantum chemical topology of electron density. *RSC Adv.* **2014**, *4*, 32415-32428.
13. Ríos-Gutiérrez, M.; Domingo, L. R. Unravelling the mysteries of the [3+2] cycloaddition reactions. *Eur. J. Org. Chem.* **2019**, 267–282.

Table S4. B3LYP/6-31G(d) total, E in a.u., and relative, ΔE in kcal·mol^{-1}, energies, in gas phase and in DCM, of the stationary points associated to the 32CA reaction of diphenyl NI **2a** with (R)-carvone **1**.

	E	ΔE	E	ΔE
	Gas phase		DCM	
NI **2a**	-610.823282		-610.830158	
**1**	-464.694795		-464.700422	
**TS-1-NI**	-1075.506125	7.5	-1075.515921	9.2
**TS-2-NI**	-1075.503157	9.4	-1075.511455	12.0
**TS-3-NI**	-1075.505129	8.1	-1075.514762	9.9
**TS-4-NI**	-1075.498053	12.6	-1075.507285	14.6
**CA-1-NI**	-1075.587420	-43.5	-1075.595255	-40.6
**CA-2-NI**	-1075.588876	-44.4	-1075.596892	-41.6
**CA-3-NI**	-1075.598526	-50.5	-1075.609230	-49.4
**CA-4-NI**	-1075.596352	-49.1	-1075.607288	-48.1

Table S5. B3LYP/6-31G(d) total, E in a.u., and relative, ΔE in kcal·mol^{-1} energies, in gas phase and in DCM, of the stationary points associated to the 32CA reaction of phenyl NO **4a** with (R)-carvone **1**.

	E	ΔE	E	ΔE
	Gas phase		DCM	
NO **4a**	-399.639884		-399.645303	
**1**	-464.694795		-464.700422	
**TS-1-NO**	-864.308459	16.5	-864.318403	17.1
**TS-2-NO**	-864.308033	16.7	-864.314973	19.3
**TS-3-NO**	-864.313564	13.2	-864.322289	14.7
**TS-4-NO**	-864.302268	20.3	-864.311629	21.4
**CA-1-NO**	-864.382811	-30.2	-864.391744	-28.9
**CA-2-NO**	-864.381682	-29.5	-864.389632	-27.6
**CA-3-NO**	-864.401742	-42.1	-864.412136	-41.7
**CA-4-NO**	-864.386731	-32.7	-864.397163	-32.3

Table S6. B3LYP/6-311G(d,p) electronic energies, E in a.u., enthalpies , H in a.u., entropies, S in cal·mol^{-1}·K^{-1}, and Gibbs free energies, G in a.u., and relative electronic energies, ΔE in kcal·mol^{-1}, enthalpies, ΔH, in kcal·mol^{-1}, entropies, ΔS in cal·mol^{-1}·K^{-1}, and Gibbs free energies, ΔG, in kcal·mol^{-1},computed at 25˚C in DCM, of the stationary points associated with the 32CA reaction of diphenyl NI **2a** with (R)-carvone **1**.

	E	ΔE	H	ΔH	S	ΔS	G	ΔG
NI **2a**	-610.978864		-610.771199		116.4		-610.826486	
**1**	-464.824178		-464.596830		104.6		-464.646535	
**TS-1-NI**	-1075.784023	11.9	-1075.348268	12.4	170.4	-50.5	-1075.429245	27.5
**TS-2-NI**	-1075.780063	14.4	-1075.344282	14.9	170.4	-50.6	-1075.425226	30.0
**TS-3-NI**	-1075.783388	12.3	-1075.347774	12.7	172.0	-49.0	-1075.429490	27.3
**TS-4-NI**	-1075.775508	17.3	-1075.339857	17.7	171.1	-49.9	-1075.421134	32.6
**CA-1-NI**	-1075.861024	-36.4	-1075.420912	-33.2	162.7	-58.3	-1075.498220	-15.8
**CA-2-NI**	-1075.862484	-37.3	-1075.422188	-34.0	161.7	-59.3	-1075.499024	-16.3
**CA-3-NI**	-1075.873967	-44.5	-1075.433590	-41.1	162.7	-58.3	-1075.510871	-23.8
**CA-4-NI**	-1075.872207	-43.4	-1075.431275	-39.7	162.5	-58.5	-1075.508484	-22.3

Table S7. B3LYP/6-311G(d,p) electronic energies, E in a.u., enthalpies , H in a.u., entropies, S in cal·mol^{-1}·K^{-1}, and Gibbs free energies, G in a.u., and relative electronic energies, ΔE in kcal·mol^{-1}, enthalpies, ΔH, in kcal·mol^{-1}, entropies, ΔS in cal·mol^{-1}·K^{-1}, and Gibbs free energies, ΔG, in kcal·mol^{-1},computed at 0˚C in DCM, of the stationary points associated with the 32CA reaction of phenyl NO **4a** with (R)-carvone **1**.

	E	ΔE	H	ΔH	S	ΔS	G	ΔG
NO **4a**	-399.747470		-399.637542		83.0		-399.673654	
**1**	-464.824178		-464.598566		100.8		-464.642443	
**TS-1-NO**	-864.541204	19.1	-864.205300	19.3	140.0	-43.8	-864.266224	31.3
**TS-2-NO**	-864.537415	21.5	-864.201396	21.8	138.4	-45.3	-864.261661	34.2
**TS-3-NO**	-864.544899	16.8	-864.208967	17.0	140.9	-42.9	-864.270290	28.7
**TS-4-NO**	-864.533812	23.7	-864.197827	24.0	138.6	-45.2	-864.258158	36.4
**CA-1-NO**	-864.610013	-24.1	-864.270593	-21.6	134.1	-49.6	-864.328986	-8.1
**CA-2-NO**	-864.607563	-22.5	-864.267849	-19.9	132.3	-51.5	-864.325420	-5.9
**CA-3-NO**	-864.629392	-36.2	-864.289598	-33.6	134.0	-49.8	-864.347906	-20.0
**CA-4-NO**	-864.614423	-26.8	-864.273873	-23.7	131.7	-52.1	-864.331191	-9.5

Table S8. B3LYP/6-311G(d,p) electronic energies, E in a.u., enthalpies, H in a.u., entropies, S in cal·mol^{-1}·K^{-1}, and Gibbs free energies, G in a.u., and relative electronic energies, ΔE in kcal·mol^{-1}, enthalpies, ΔH, in kcal·mol^{-1}, entropies, ΔS in cal·mol^{-1}·K^{-1}, and Gibbs free energies, ΔG, in kcal·mol^{-1},computed at 25˚C in DCM, of the stationary points associated with the 32CA reaction of simplest NI **8** and simplest NO **9** with ethylene **6**.

	E	ΔE	H	ΔH	S	ΔS	G	ΔG
NI **8**	-148.747639		-148.711645		58.6		-148.739489	
**6**	-78.615040		-78.560324		55.1		-78.586490	
**TS-NI**	-227.347276	9.7	-227.255280	10.5	74.0	-39.7	-227.290426	22.3
**CA-NI**	-227.452296	-56.2	-227.353568	-51.2	67.8	-45.9	-227.385766	-37.5
NO **9**	-168.627498		-168.603733		50.6		-168.627770	
**TS-NO**	-247.218978	14.8	-247.138943	15.8	73.2	-32.5	-247.173700	25.5
**CA-NO**	-247.304117	-38.6	-247.219523	-34.8	65.5	-40.2	-247.250649	-22.8

Table S9. B3LYP/6-311G(d,p) enthalpies, H in a.u., entropies, S in cal·mol^{-1}·K^{-1}, and Gibbs free energies, G in a.u., and relative enthalpies, ΔH, in kcal·mol^{-1}, entropies, ΔS in cal·mol^{-1}·K^{-1}, and Gibbs free energies, ΔG, in kcal·mol^{-1}, computed at 0˚C in DCM, of the stationary points associated with the 32CA reaction of simplest NO **9** with ethylene **6**.

	H	ΔH	S	ΔS	G	ΔG
NO **9**	-168.604214		49.5		-168.625775	
**6**	-78.560716		54.2		-78.584313	
**TS-NO**	-247.139757	15.8	71.4	-32.4	-247.170821	24.6
**CA-NO**	-247.220091	-34.6	64.3	-39.5	-247.248064	-23.8
